# Spatiotemporal Dynamics of the Eco-Physiological Characteristics of *Picea schrenkiana* in the Tianshan Mountains and Its Adaptive Mechanisms

**DOI:** 10.3390/plants15081199

**Published:** 2026-04-14

**Authors:** Ruixi Li, Lu Gong, Xue Wu, Kejie Yin, Yihu Niu, Xiaonan Sun, Peryzat Abay, Fan Tian

**Affiliations:** 1 College of Ecology and Environment, Xinjiang University, Urumqi 830017, China; 2 Key Laboratory of Oasis Ecology of Education Ministry, Urumqi 830017, China; 3 Xinjiang Jinghe Observation and Research Station of Temperate Desert Ecosystem, Ministry of Education, Jinghe 830017, China

**Keywords:** eco-physiological characteristics, arid mountainous forest ecosystems, *Picea schrenkiana*, environmental factors, seasonal variations

## Abstract

Trees in arid mountainous forests adapt to seasonal water variability through dynamic eco-physiological adjustments. This study investigated the spatiotemporal dynamics and environmental drivers of such adaptations in *Picea schrenkiana* Fisch. et Mey, a keystone conifer in China’s Tianshan Mountains. We monitored key indicators—including osmoregulatory substances, antioxidant enzyme activities, and stoichiometric traits—across three regions (eastern, central, western) and three seasons (spring, summer, autumn) during the 2023 growing season. The results revealed significant seasonal shifts in all the measured traits (*p* < 0.05). Spring was characterized by high carbon allocation toward soluble sugars and starch, supporting growth; summer triggered elevated antioxidant enzyme activities to mitigate oxidative stress; and autumn favored nitrogen accumulation and proline synthesis, indicating preparatory storage for winter. Soil factors were primarily positively associated with antioxidant enzyme activity (path coefficient = 0.51; *p* < 0.001), whereas microenvironmental factors were more complex and often negatively correlated. The partial least squares path model confirmed that osmoregulatory substances centrally link stoichiometric adjustments with antioxidant defense, revealing an integrated physiological strategy. These findings elucidate the mechanism underlying the resilience of *P. schrenkiana* in arid highlands and provide a framework for its conservation under environmental change.

## 1. Introduction

Under the severe challenges of global climate change, both meteorological and soil factors significantly influence trees within forest ecosystems [1]. As major carbon sinks and critical water regulation systems, forests face unprecedented threats to their ecological functions [2]. The interaction between environmental pressure and internal regulatory mechanisms in trees leads to changes in plant eco-physiological characteristics, which reflect their adaptive capacities [1,3]. Therefore, in-depth investigations into the spatiotemporal patterns of these changes in forest trees and elucidation of the underlying adaptation and regulatory mechanisms have become prominent topics in ecological research.

The adaptive responses of plant eco-physiological characteristics demonstrate high sensitivity to environmental changes and complex adaptive strategies. The flow, transport, and transformation of substances depend on soil water movement, and the soil water status serves as the direct driving force for root water uptake. When soil moisture decreases, the water acquisition capacity of trees increases by increasing the root-to-shoot ratio [4] or adjusting vessel structures (such as the “evaporation-driven hydraulic redistribution” mechanism observed in *Populus tomentosa*) [5]. Light intensity and temperature are key factors influencing the eco-physiological characteristics of trees [6], directly affecting water use by regulating stomatal opening and closing, transpiration rates, and photosynthetic efficiency [7]. Rising temperatures accelerate transpiration, but under extreme heat, trees may close their stomata to reduce water loss [8]. These complex eco-physiological responses reveal the adaptability and survival capacity of trees in the face of environmental stress [9], demonstrating how trees regulate their internal water balance at the micro level, thereby influencing ecosystem stability and diversity at the macro level.

The intrinsic regulatory adaptability of tree eco-physiological characteristics involves multilevel control mechanisms, including osmotic adjustment, antioxidant defense, and resource allocation strategies (stoichiometric regulation) [5,7]. The synergistic action of these mechanisms enables trees to cope with seasonal changes in environmental factors while maintaining water balance and stabilizing their physiological functions. Stoichiometric regulation enables trees to effectively regulate osmotic adjustment substances, structural traits, and key enzymes in response to seasonal environmental fluctuations [10]. Under water-deficient conditions, trees optimize carbon allocation by reducing lignin production for structural growth and increasing the synthesis of soluble sugars for osmotic adjustment [5] while ensuring the continuation of photosynthesis to provide essential material support for plant growth and development. Osmotic adjustment substances (such as soluble proteins, soluble sugars, and proline) are considered key physiological responses in the osmotic regulatory system of forest plants to cope with stress, helping trees maintain cell turgor pressure under low water potential and resist environmental pressures [7]. The antioxidant defense system, which is composed of enzymes such as superoxide dismutase, peroxidase, and catalase, maintains the reactive oxygen species balance [10], enhances tree resistance, and ensures cellular integrity and function. These adjustments in eco-physiological characteristics not only ensure tree survival under changing environments but also directly influence growth patterns by altering resource allocation and energy balance.

However, how these intrinsic regulatory mechanisms are integrated and expressed across both temporal (seasonal) and spatial (regional) gradients in key forest tree species remains poorly understood, particularly in sensitive arid mountain ecosystems. As a widely distributed dominant and sole constructive species in the Tianshan Mountains of Xinjiang, China, *Picea schrenkiana* adapts to environmental changes across different seasons by altering its eco-physiological characteristics [11]. This adaptive strategy fully reflects its spatiotemporal adaptability to mountain forest ecosystems in arid regions. Its root system is distributed mainly in the 0–60 cm soil layer [12], indirectly reflecting the belowground distribution pattern typical of temperate forest tree species. *P. schrenkiana* plays irreplaceable ecological roles in water conservation, soil retention, and the maintenance of biodiversity [13]. In this study, the spatiotemporal variability and correlations of eco-physiological characteristics in *P. schrenkiana* were investigated, with the goal of identifying the main environmental factors regulating these traits. This research addresses the following questions: (1) How do the eco-physiological characteristics of *P. schrenkiana* respond to the dynamics of environmental factors? (2) What key eco-physiological adaptive strategies does *P. schrenkiana* employ under spatiotemporal dynamics?

## 2. Results

### 2.1. Environmental Factors

**Figure 1:**
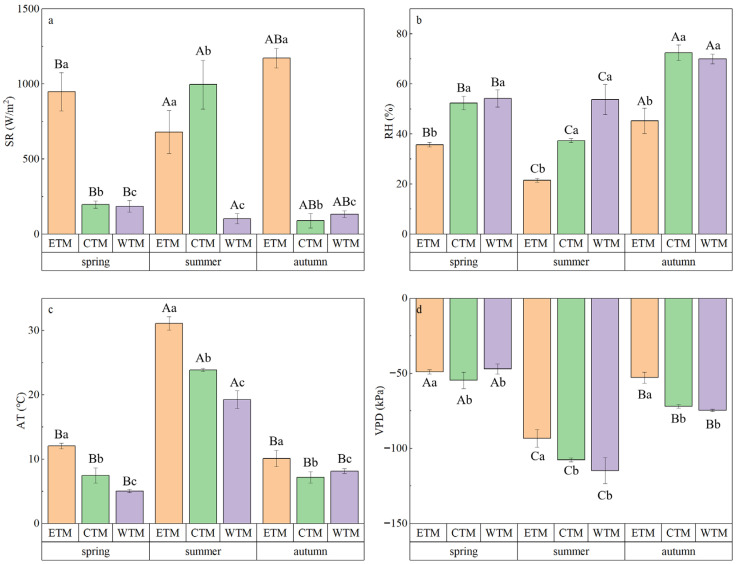
Spatiotemporal variations in microenvironmental factors: (**a**) solar radiation (SR), (**b**) relative humidity (RH), (**c**) air temperature (AT), and (**d**) vapor pressure deficit (VPD). Data were analyzed by two-way ANOVA (season × region). Different uppercase letters indicate significant differences among seasons when data from all regions are pooled. Different lowercase letters indicate significant differences among regions when data from all seasons are pooled (post hoc Fisher’s LSD test, *p* < 0.05). Data are presented as mean ± standard error (SE). Sampling rounds correspond to spring, summer, and autumn sampling dates, respectively.

All the microenvironmental factors except solar radiation significantly differed across the sampling sites and sampling dates (*p* < 0.001; Figure 1, Table S1). The detailed seasonal microenvironmental conditions—including air temperature, relative humidity, solar radiation and vapor pressure deficit—for each sampling station are summarized in Table S2. Significant spatiotemporal interactions were also detected (*p* < 0.05). Among them, the solar radiation in the eastern Tianshan Mountains was significantly greater than that in the other regions during the spring and autumn sampling rounds (see Section 4.2 for details on the sampling rounds), whereas the central Tianshan Mountains received greater solar radiation during the summer sampling rounds. Additionally, the vapor pressure deficit was relatively low in the central Tianshan Mountains during spring sampling rounds and tended to decrease from east to west during both summer and autumn sampling rounds.

**Figure 2:**
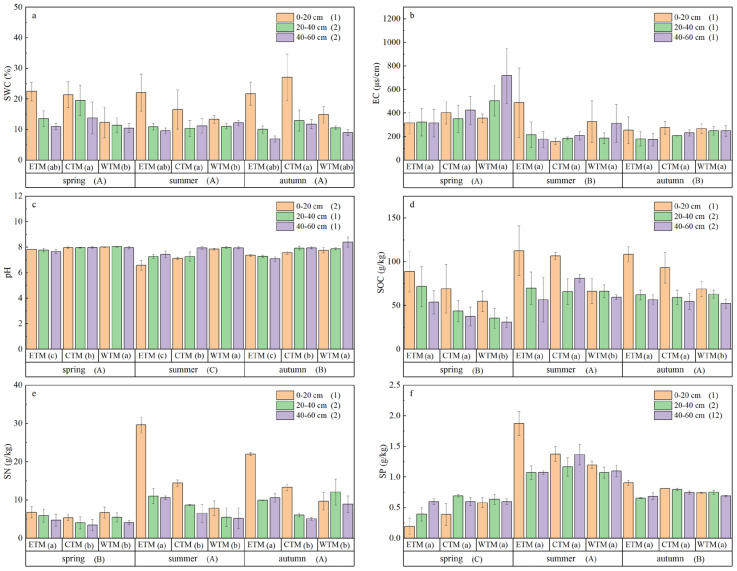
Three-way ANOVA (season × region × soil depth) of soil factors: Variations in (**a**) soil water content (SWC), (**b**) electrical conductivity (EC), (**c**) pH, (**d**) soil organic carbon (SOC), (**e**) total soil nitrogen (TN), and (**f**) total soil phosphorus (TP). Different uppercase letters indicate significant differences among seasons when data from all regions and soil depths are pooled. Different lowercase letters indicate significant differences among regions when data from all seasons and soil depths are pooled. Different numerals indicate significant differences among soil depths when data from all seasons and regions are pooled (post hoc Fisher’s LSD test, *p* < 0.05). Data are presented as mean ± standard error (SE).

Significant spatiotemporal heterogeneity was observed for all key soil factors (Figure 2, Table S3). The soil water content varied significantly by region and soil depth, with higher values in the central Tianshan Mountains and in the surface layer (0–20 cm). Soil pH, soil organic carbon, and total nitrogen significantly differed across seasons, regions, and soil depths (*p* < 0.05). In contrast, significant seasonal variations in soil electrical conductivity and total phosphorus content were detected. With respect to the seasonal patterns, the soil pH was significantly greater in spring than in summer and autumn. Conversely, the total nitrogen content was lower in spring than in the other seasons. The total phosphorus content followed a decreasing gradient: summer > autumn > spring. Furthermore, significant interactive effects of season × region were observed for soil pH, total nitrogen, and total phosphorus (*p* < 0.05; Table S3), highlighting that their dynamics were jointly governed by temporal and spatial factors.

### 2.2. Stoichiometric Traits

**Figure 3:**
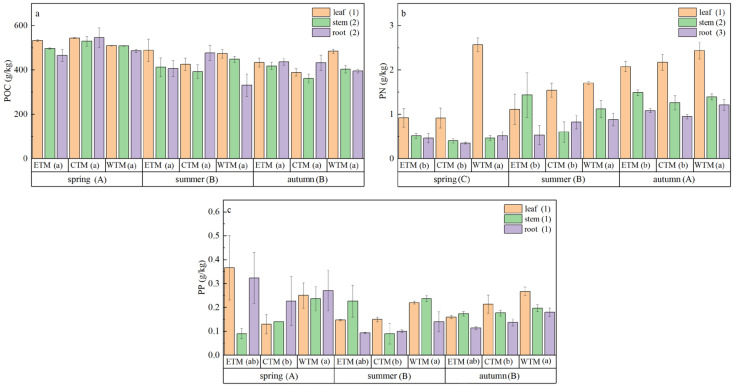
Variations in ecological stoichiometric traits of *P. schrenkiana* across seasons, regions, and organs: (**a**) plant organic carbon (POC), (**b**) plant total nitrogen (PN), and (**c**) plant total phosphorus (PP). Different uppercase letters indicate significant differences among seasons when data from all regions and soil depths are pooled. Different lowercase letters indicate significant differences among regions when data from all seasons and soil depths are pooled. Different numerals indicate significant differences among organs when data from all seasons and regions are pooled (post hoc Fisher’s LSD test, *p* < 0.05). Data are presented as mean ± standard error (SE).

The stoichiometric traits of *P. schrenkiana* significantly varied across seasons, regions, and plant organs (Figure 3, Table S4). Seasonally, the plant organic carbon and total phosphorus contents were significantly higher in spring than in summer and autumn. In contrast, the total nitrogen content was significantly higher in autumn than in spring and summer (*p* < 0.05). Across different geographical regions, only the total nitrogen and total phosphorus contents significantly differed (*p* < 0.05). Specifically, the total nitrogen content in the western Tianshan Mountains was significantly greater than that in the eastern and central Tianshan Mountains. Among different plant organs, a highly significant difference was observed only for total nitrogen content (*p* < 0.001), with leaves containing significantly higher levels than roots and stems did. With respect to the interactive effects of spatiotemporal factors and organs, no significant differences in stoichiometric traits were found under the sole influence of spatiotemporal conditions (i.e., the interaction between season and region) (*p* > 0.05; Table S4). Finally, under the three-way interaction of season, region, and organ, only the total nitrogen content among all the stoichiometric traits significantly differed (*p* < 0.05; Table S4).

### 2.3. Osmoregulatory Substances

**Figure 4:**
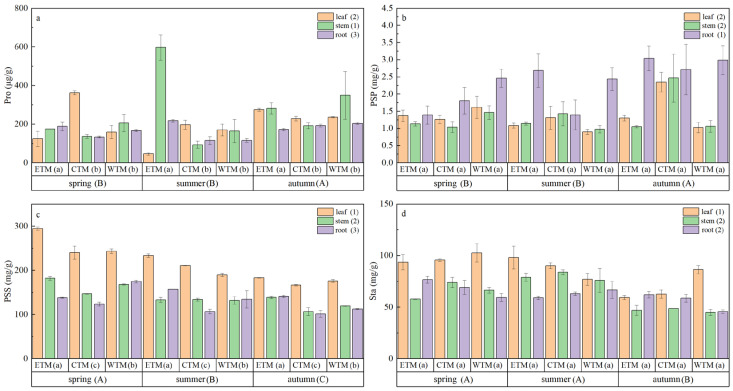
Variations in osmoregulatory substances of *P. schrenkiana* across seasons, regions, and organs: (**a**) Proline (Pro), (**b**) Soluble Protein (PSP), (**c**) Soluble Sugar (PSS), and (**d**) Starch (Sta). Different uppercase letters indicate significant differences among seasons when data from all regions and soil depths are pooled. Different lowercase letters indicate significant differences among regions when data from all seasons and soil depths are pooled. Different numerals indicate significant differences among organs when data from all seasons and regions are pooled (post hoc Fisher’s LSD test, *p* < 0.05). Data are presented as mean ± standard error (SE).

As shown in Figure 4 and Table S5, the osmoregulatory substances in *P. schrenkiana* significantly varied across seasons and plant organs (*p* < 0.05). Specifically, the soluble sugar and starch contents were significantly higher in spring than in summer and autumn, whereas the proline and soluble protein contents peaked in autumn. In terms of organ-specific distribution, leaves presented higher soluble sugar and starch contents than roots and stems did, whereas roots accumulated significantly more soluble protein. Among the different geographical regions, only the proline and soluble sugar levels significantly differed (*p* < 0.05). Three-way ANOVA revealed that the main effects of both season and region significantly influenced the contents of proline, soluble protein, and soluble sugar (*p* < 0.05; Table S5). Notably, the three-way interaction effect of season, region, and organ had a highly significant effect on the proline and soluble sugar contents (*p* < 0.001; Table S5).

### 2.4. Antioxidant Enzyme Activities

**Figure 5:**
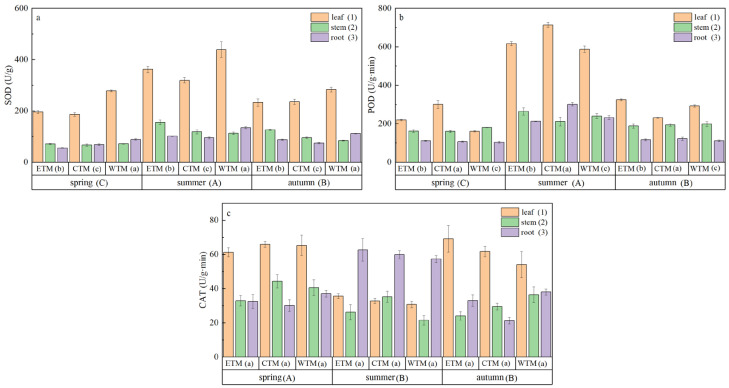
Variations in (**a**) SOD (superoxide dismutase), (**b**) POD (peroxidase), (**c**) CAT (catalase activities) in *P. schrenkiana* across different seasons, regions, and plant organs (leaves, stems, roots). Different uppercase letters indicate significant differences among seasons when data from all regions and soil depths are pooled. Different lowercase letters indicate significant differences among regions when data from all seasons and soil depths are pooled. Different numerals indicate significant differences among organs when data from all seasons and regions are pooled (post hoc Fisher’s LSD test, *p* < 0.05). Data are presented as mean ± standard error (SE).

As shown in Figure 5 and Table S6, plant antioxidant enzyme activities significantly varied across seasons, regions, and organs. Specifically, a seasonal analysis revealed that the activities of superoxide dismutase and peroxidase were greater in summer than in spring and autumn. In terms of organ-specific differences, compared with the roots and stems, the leaves presented significantly greater superoxide dismutase activity and peroxidase activity. Among the regions, only the levels of superoxide dismutase and peroxidase significantly varied (*p* < 0.05). Finally, the three-way interactions among season, region, and organ were significant only for peroxidase activity (*p* < 0.001; Table S6).

### 2.5. Relationships Between Eco-Physiological Traits and Environmental Factors

**Figure 6:**
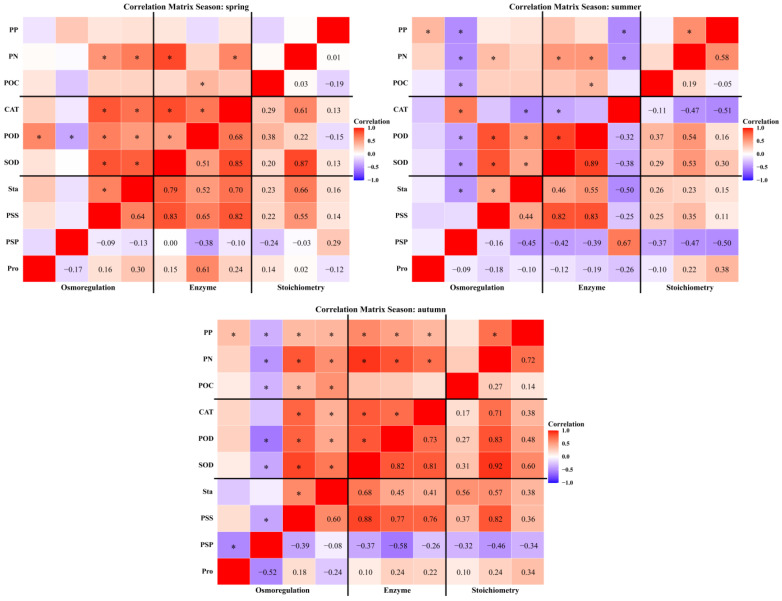
Correlation heatmaps between plant eco-physiological characteristics (stoichiometric traits, osmoregulatory substances, and antioxidant enzymatic activities) across seasons. *: *p* < 0.05.

**Figure 7:**
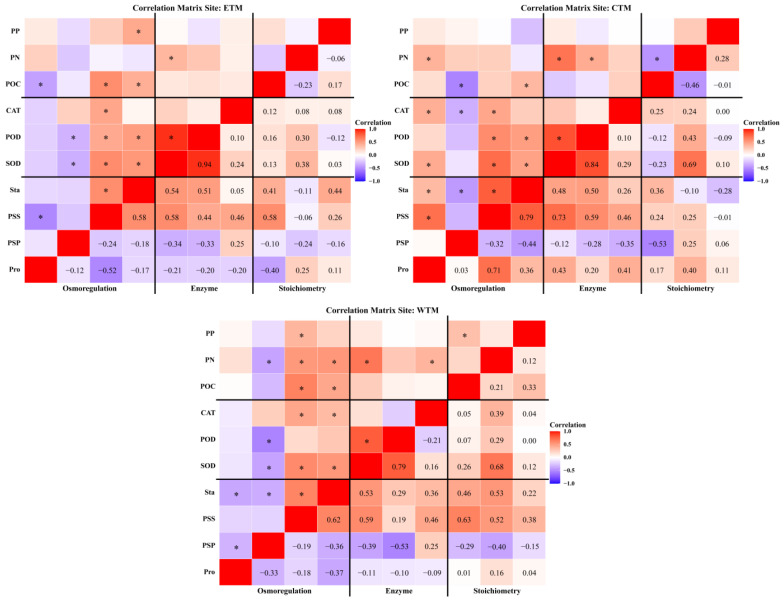
Correlation heatmaps between plant eco-physiological characteristics (stoichiometric traits, osmoregulatory substances, and antioxidant enzymatic activities) across regions. *: *p* < 0.05.

**Figure 8:**
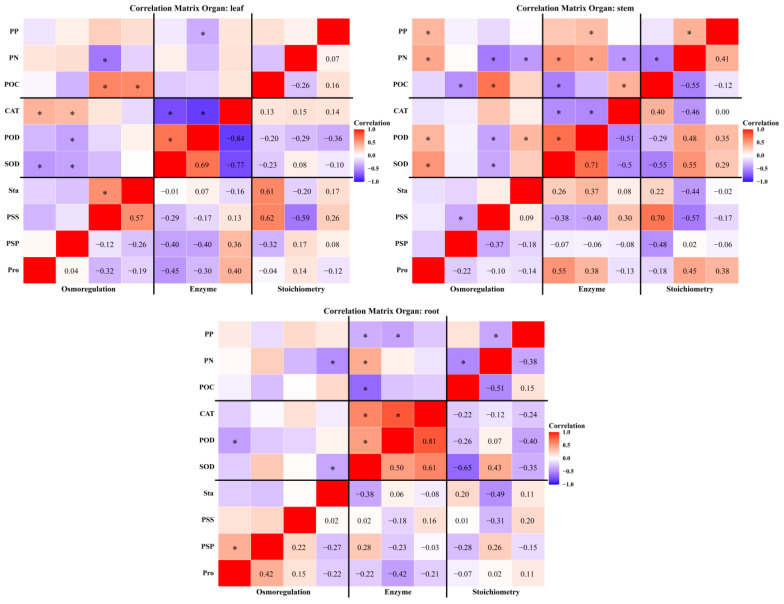
Correlation heatmaps between plant eco-physiological characteristics (stoichiometric traits, osmoregulatory substances, and antioxidant enzymatic activities) across plant organs. *: *p* < 0.05.

Significant correlations were observed among plant osmotic adjustment substances, antioxidant enzyme activities, and stoichiometric traits, with these relationships varying by season, region, and plant organ. On a seasonal scale (Figure 6), soluble sugars and starch were significantly positively correlated with various antioxidant enzyme activities and stoichiometric traits in both spring and autumn. In autumn, plant total nitrogen and total phosphorus were also positively correlated with enzyme activity. In summer, soluble sugars and starch were primarily positively correlated with superoxide dismutase and peroxidase activity. Regionally (Figure 7), in the eastern and central Tianshan Mountains, soluble sugar and starch correlated positively with superoxide dismutase and peroxidase. In the western Tianshan Mountains, these substances were positively correlated with superoxide dismutase, catalase, total nitrogen, and total phosphorus. At the organ level (Figure 8), leaf soluble protein was negatively correlated with superoxide dismutase and peroxidase activity but positively correlated with catalase activity. Stem proline was positively correlated with superoxide dismutase activity, peroxidase activity, total nitrogen content, and total phosphorus content. Root superoxide dismutase activity was negatively correlated with organic carbon and total phosphorus but positively correlated with total nitrogen.

**Figure 9:**
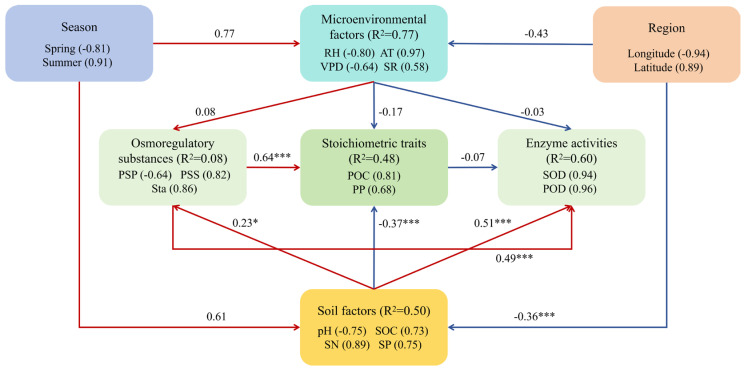
Eco-physiological characteristics regulatory pathways in *P. schrenkiana* driven by soil factors, microenvironmental factors, and spatiotemporal gradients within the PLS–PM framework. Solid red and blue arrows denote significant positive and negative path coefficients, respectively (*** *p* < 0.001, * *p* < 0.05). Model parameters were bootstrapped (n = 500 iterations) with goodness-of-fit indices confirming robustness (Average R^2^ = 0.49, GoF = 0.57).

The results of the PLS–PM analysis (Figure 9; Table S7) demonstrated that environmental factors were linked to ecosystem processes through multiple pathways: region showed a significant direct negative association with soil factors (path coefficient = −0.36, *p* < 0.001), whereas soil factors were strongly positively correlated with plant antioxidant enzyme activities (path coefficient = 0.51, *p* < 0.001). The effect decomposition analysis (Table S7) revealed that the influence of soil factors on antioxidant enzyme activities was primarily direct (direct effect = 0.51), with minimal indirect contributions. Similarly, plant osmoregulatory substances strongly affected both stoichiometric traits (direct effect = 0.64, *p* < 0.001) and antioxidant enzyme activities (direct effect = 0.49, *p* < 0.001). Although the pathways from season to microenvironmental factors and region to microenvironmental factors did not reach statistical significance, their substantial coefficients suggest potential ecological importance.

## 3. Discussion

The physiological ecology of *P. schrenkiana* across the Tianshan Mountains remains insufficiently explored, particularly in terms of its spatiotemporal dynamics, sensitivity to environmental changes, and underlying physiological constraints. Our findings reveal that the eco-physiological characteristics of *P. schrenkiana*—including stoichiometric traits, osmoregulatory substances, and antioxidant enzyme activities—exhibit significant seasonal variations. Furthermore, these traits were significantly influenced by complex spatiotemporal interactions, highlighting that the plant’s physiological responses are not governed by a single factor but by the interplay of time and space. Critically, specific osmoregulatory substances were significantly correlated with antioxidant enzyme activity, which varied by season. The PLS–PM identified potential pathways of association: the region showed a significant direct negative association with soil factors, whereas soil factors were positively correlated with plant antioxidant enzyme activities. Additionally, plant osmoregulatory substances were strongly associated with both stoichiometric traits and antioxidant enzyme activities. These findings collectively underscore a sophisticated and multifaceted adaptive strategy in *P. schrenkiana*.

### 3.1. An Integrated Spatiotemporal Strategy: Coordination Among Stoichiometry, Osmotic Adjustment, and Antioxidant Defense in P. schrenkiana

Our findings reveal that *P. schrenkiana* employs a sophisticated, multitiered physiological strategy to manage spatiotemporal heterogeneity. This strategy is characterized by tight coordination and dynamic trade-offs among elemental stoichiometry, osmotic adjustment, and antioxidant enzyme activity rather than by independent responses of these traits. The plant’s physiological profile is not static but is fine-tuned through significant interactions among season, region, and organ, demonstrating a high degree of phenotypic integration.

At the core of this integration, osmoregulatory substances appear to serve as a central component associated with resource allocation and stress defense. Soluble sugars and starch accumulated in spring serve dual purposes: supporting cellular turgor for growth and providing metabolic substrates for subsequent antioxidant synthesis [14]. Conversely, the marked seasonal shift toward proline and soluble protein accumulation in autumn represents a strategic reallocation of resources from growth-promoting solutes to cryoprotective compounds [15]. These osmoregulatory dynamics were significantly correlated with antioxidant enzyme activity, suggesting a synergistic effect. This synergy is evidenced by positive correlations, suggesting that osmolytes may function in parallel as both protectants and energy sources for the antioxidant system, while proline accumulation is associated with enhanced oxidative stress defense [14].

This coordinated response is fundamentally underpinned by stoichiometric flexibility, particularly in terms of nitrogen metabolism. The significant accumulation of total nitrogen across organs in autumn indicates a crucial storage strategy to fuel future growth—an “investment in the future” critical for survival in seasonal ecosystems. At the organ level, robust support for the “Optimal Partitioning Theory” is clear, with leaves maintaining the highest nitrogen content to maximize photosynthetic carbon gain [16]. Most importantly, the significant three-way interaction (season × region × organ) solely for total nitrogen content established it as the central metabolic hub through which *P. schrenkiana* integrates complex spatiotemporal signals and internal functional demands. The positive correlations observed between stem proline, regional osmotic substances, and total nitrogen and phosphorus further reflect a tight coupling between nutrient metabolism and the synthesis of organic solutes.

In addition to the coordination between osmoregulatory substances and antioxidant enzymes, the seasonal dynamics of nitrogen and phosphorus concentrations in different organs play a critical physiological role in stress adaptation. The significant accumulation of total nitrogen in stems and roots during autumn reflects a storage strategy that supports early spring growth when soil nitrogen availability is low—a common adaptive trait in perennial conifers facing prolonged winter dormancy. In contrast, the seasonal shift in P concentrations, particularly the decline in leaves during summer, may be linked to the reallocation of phosphorus to support metabolic processes such as ATP synthesis and antioxidant enzyme function under oxidative stress. The organ-specific patterns of nitrogen and phosphorus further suggest that *P. schrenkiana* optimizes nutrient allocation according to functional demands: leaves prioritize nitrogen for photosynthetic carbon gain, whereas stems and roots serve as storage reservoirs. These stoichiometric adjustments, when considered alongside osmotic and antioxidant responses, form an integrated nutritional and metabolic strategy for coping with seasonal environmental fluctuations.

The antioxidant defense system exhibits a complementary temporal hierarchy, acting as a rapid-response module to immediate stressors. The elevated activities of superoxide dismutase and peroxidase during summer constitute a frontline defense against reactive oxygen species generated by concurrent abiotic stresses [17]. This enzymatic response is highly context dependent, as shown by the pronounced season × organ interactions, where leaves—the primary sites of photooxidative stress—show the greatest responsiveness. Notably, the three-way interaction was highly significant only for peroxidase activity, implying that this downstream enzyme possesses the most complex regulatory mechanism and is capable of precisely integrating seasonal, regional, and organ-specific signals for targeted defense [18].

The PLS–PM is consistent with this integrated network. These findings suggest that osmoregulatory substances are strongly directly associated with both stoichiometric traits and antioxidant enzyme activities. This pathway analysis is consistent with the view of osmotic adjustment not only as a passive stress response but also as potentially playing an integrative role that may be related to plant nutrient homeostasis and the coordination of the oxidative stress defense system. This intricate coordination among stoichiometric plasticity, targeted osmotic adjustment, and temporally hierarchical antioxidant defense encapsulates the cohesive physiological strategy that enables *P. schrenkiana* to thrive across the heterogeneous landscapes of the Tianshan Mountains.

### 3.2. Environmental Drivers and Their Hierarchical Influences on Physiological Processes

The results of the PLS–PM analysis revealed that plant antioxidant enzyme activities were conjointly associated with a complex interplay of soil and microenvironmental factors. Soil factors had a significant positive effect (path coefficient = 0.51; *p* < 0.001), whereas microenvironmental factors had a comparable positive effect (path coefficient = 0.31), underscoring the multidimensional nature of environmental control over plant physiology. Soil factors—including moisture, organic carbon, nitrogen, and phosphorus—are strongly associated with enzymatic profiles, potentially through resource provision and stress signaling pathways [19,20]. Moreover, microenvironmental factors such as solar radiation and the vapor pressure deficit were strongly associated with plant stress status.

Critically, the effects of these environmental drivers were not static but exhibited pronounced spatiotemporal heterogeneity. Seasonal dynamics were a primary determinant, with microenvironmental factors showing strong seasonal dependence (the path coefficient from season to microenvironmental factors was 0.34, although not statistically significant, suggesting potential ecological importance). This finding is consistent with the observed peak in solar radiation and associated abiotic stress during summer, which coincided with the maximal activities of superoxide dismutase and peroxidase, suggesting an acute defensive association with accelerated reactive oxygen species generation [17]. Conversely, regional gradients also shaped environmental patterns, as evidenced by the notable negative influence of region on soil factors (path coefficient = −0.36, *p* < 0.001), leading to distinct climatic regimes across the transect.

This spatiotemporal heterogeneity was further amplified through interactive effects on both the environment and the plant. Significant season × region interactions were observed for key soil properties (pH, total nitrogen, and total phosphorus), indicating that the soil conditions themselves vary dynamically across time and space. Consequently, the same nominal environmental factor could transmit distinct signals to the plant depending on the context. For instance, the coupling of higher spring solar radiation in the eastern region with elevated surface soil moisture may create a unique microenvironment of alternating wet–dry cycles, differentially regulating nutrient uptake and oxidative stress.

The physiological response of the plant to this complex environmental matrix is profoundly organ-specific. The significant season × organ interactions for nearly all osmoregulatory substances and antioxidant enzymes highlight that environmental signals are filtered and interpreted differently by leaves, stems, and roots. Leaves, as the primary interface with atmospheric stressors, exhibited consistently increased superoxide dismutase and peroxidase activities, confirming their frontline role in mitigating photooxidative damage [18].

In summary, our findings delineate a hierarchical environmental regulatory network: large-scale seasonal and regional factors define the overarching spatiotemporal patterns of microenvironmental and soil conditions. These conditions, in turn, interact synergistically (e.g., season × region) to create a mosaic of microenvironmental challenges, which are perceived and resolved in an organ-specific manner (e.g., season × organ), leading to the finely tuned, integrated physiological responses observed in *P. schrenkiana*. This cascade of interactions underscores the complexity of predicting physiological outcomes under climate change, where shifts in one driver can reverberate through this entire network.

### 3.3. Physiological Integration and Adaptive Implications Under Climate Change

*P. schrenkiana* adapts to spatiotemporal heterogeneity through a cohesive strategy that integrates metabolic coordination, rapid enzymatic responses, and multidimensional phenotypic plasticity. This integration is fundamentally rooted in the coordination of carbon and nitrogen metabolism, where osmoregulatory substances and antioxidant enzymes show synergistic associations under stress [21]. Soluble sugars and starch may function as osmoregulants and potential energy substrates for the synthesis and function of antioxidant enzymes such as superoxide dismutase and peroxidase, with their correlations varying seasonally; in summer, they were primarily correlated with superoxide dismutase and peroxidase, whereas in spring and autumn, they were significantly positively correlated with a broader range of antioxidant enzyme activities and stoichiometric traits (*p* < 0.001) [14,22]. This metabolic linkage enables dynamic resource allocation aligned with seasonal environmental demands. Comparable patterns of carbon–nitrogen metabolic coordination have been observed in other conifers. For instance, studies on *Picea crassifolia* in the Helan Mountains revealed that foliar carbon (C) and nitrogen (N) stoichiometry is tightly coupled with soil nutrient availability and that elevational shifts in resource allocation reflect a similar integration of carbon and nitrogen metabolism in response to spatial environmental gradients [23]. Similarly, research on four conifer species (*Picea crassifolia*, *Abies fabri*, *Pinus tabuliformis*, and *Larix gmelinii*) on the northeastern Tibetan Plateau has demonstrated that leaf C:N:P stoichiometry exhibits pronounced seasonal variations, with soluble sugars and nitrogen pools covarying to meet phenological demands, reinforcing the view that metabolic coordination between carbon and nitrogen is a conserved adaptive strategy across diverse conifer taxa [24].

The plant’s physiological responses suggest a temporal and functional structure in terms of timing and specificity. Antioxidant enzymes (e.g., superoxide dismutase and peroxidase) function as rapid-response modules, showing highly significant variations (*p* < 0.001) under season–organ interactions, thereby countering short-term stress fluctuations [17]. In contrast, osmotic adjustments (e.g., proline and soluble protein accumulation) represent a slower, more sustained investment, often peaking in autumn as a compensatory mechanism for cumulative stressors such as drought and cold [15,25]. This temporal decoupling optimizes energy use under transient versus chronic stress. This temporal partitioning of antioxidant and osmotic adjustments is consistent with findings in other *Picea species*. In *Picea abies*, studies on seedling antioxidant capacity under abiotic stress (e.g., cold plasma and electromagnetic field treatments) revealed that rapid changes in antioxidant enzyme activities serve as an immediate defense, whereas longer-term adjustments in osmolyte pools reflect sustained acclimation [3]. Similarly, in *Pinus massoniana*, the process of winter reddening involves distinct temporal dynamics of antioxidant enzymes and osmotic substances, supporting the idea that conifers usually decouple rapid enzymatic responses from slower osmotic adjustments to cope with seasonal stress [4].

Spatially, *P. schrenkiana* demonstrates remarkable phenotypic plasticity across regions and organs—a key adaptive trait in heterogeneous landscapes. Compared with roots and stems, leaves exhibit distinct physiological profiles, with organ-specific patterns of osmoregulatory substances and antioxidant enzyme activities [16]. Significant regional differences in plant total nitrogen, total phosphorus, and certain osmoregulants further indicate that populations fine-tune their physiology to local soil and climate conditions. Critically, the pervasive interactive effects (season × region, season × organ, region × organ) on multiple traits are consistent with the view that the plant’s physiology is not governed by isolated factors but reflects the integrated effects of spatiotemporal drivers.

However, climate change threatens to disrupt this finely tuned adaptive system. The significant season × region interactions observed for both soil properties (e.g., pH and total nitrogen) and key plant traits (e.g., total nitrogen content) suggest that converging temporal and spatial stressors may push physiological integration beyond its sustainable limits [26]. As microenvironmental extremes and soil resource heterogeneity intensify, the environmental–plant linkages revealed by PLS–PM analysis may be disrupted, potentially overwhelming the coordinated defense network.

### 3.4. Limitations and Future Perspectives

Several limitations of this study should be acknowledged. First, the study was conducted over a single year, limiting our ability to capture the interannual variability in physiological responses. Second, measurements were confined to discrete seasonal time points, potentially missing fine-scale dynamics during seasonal transitions. Third, although PLS–PM was used to explore environmental–plant associations, the correlative nature of the analysis precludes definitive causal inference. Fourth, the stoichiometry was assessed at the whole-organ level; future studies should consider subcellular or tissue-specific distributions. Finally, the sampling was restricted to four sites along a regional transect; expanding the spatial coverage would enhance generalizability. Addressing these limitations is essential for predicting the resilience of *P. schrenkiana* under future climate scenarios. Future research should integrate transcriptomic and molecular approaches to elucidate the genetic and regulatory networks underlying the spatiotemporal physiological integration documented herein.

## 4. Materials and Methods

### 4.1. Study Site

This study was conducted across a pronounced climatic gradient on the northern slopes of the Tianshan Mountains in Xinjiang, China. Sampling stations were established in three geographically distinct regions: the western (WTM: Wusu city), central (CTM: Urumqi County), and eastern (ETM: Barkol County) sections (Table 1). This entire study area experiences a temperate continental climate, characterized by a long, severe winter and a relatively short warm season. Spatio-climatic analysis of the data (2000–2024, NASA POWER) revealed a strong west-to-east environmental gradient, which formed the basis for our investigation into spatial physiological variation (Table 2). Specifically, precipitation decreases markedly from west (310 mm) to east (63 mm), with most precipitation concentrated in summer. The central and western ranges intercept moisture from the westerlies, and the eastern region is drier because of the influence of surrounding deserts. Thermally, the mean annual temperature in the east is 2–3 °C higher than that in the west, where glacial effects exert a cooling influence. This gradient in water and heat availability is expected to be a key driver of differential physiological adaptation in *P. schrenkiana*. The soils across all the sampling stations are uniformly classified as slightly acidic mountain gray–brown forest soil, which is the dominant soil type in the mid- to high-elevation forest belt of the northern Tianshan Mountains. The vegetation is predominantly boreal temperate coniferous forest, with *P. schrenkiana* forming dominant stands on mid- to high-elevation shady slopes across the study area. All the sampling plots were established in natural, monospecific, mature coniferous forests dominated exclusively by *P. schrenkiana*. No other tree species were present in the sampled stands. The mean diameter at breast height (DBH) for the dominant trees ranged from 23 to 25 cm, and the mean height (H) ranged from 8 to 10 m across the three regions (Table 2).

### 4.2. Experimental Design and Sampling

This study employed a full factorial design encompassing three spatial regions (east, central, and west) and three temporal sampling rounds (spring, summer, and autumn). Within each region, three 10 m × 10 m permanent plots were established on shady slopes at comparable elevations dominated by *P. schrenkiana*, resulting in a total of 9 plots. Sampling campaigns were conducted once per season, in April (spring), July (summer), and October (autumn) of 2023, resulting in 9 plots × 3 sampling rounds = 27 independent sampling events. For each sampling event, three healthy, dominant *P. schrenkiana* trees were sampled per plot. Thus, a total of 27 trees were sampled per region (3 plots × 3 trees × 3 sampling rounds). The same individual trees were repeatedly measured across the three seasons to enable direct temporal comparisons at the individual level. All environmental and plant physiological measurements were performed during these three sampling events. The terms “spring,” “summer,” and “autumn” are used herein solely as labels to distinguish the three sampling dates; they do not imply that the microenvironmental conditions measured on these single days represent the climatic averages of these seasons.

### 4.3. Plant Sample Collection and Processing

During each event, three representative, healthy *P. schrenkiana* trees were selected within each plot as biological replicates (n = 3 per plot per season). The same three trees per plot were repeatedly sampled across all three seasonal campaigns to maintain consistency for seasonal comparisons. Mature, sun-exposed branches were collected from each tree using branch clippers. On site, stems and leaves were manually separated. Fine roots were carefully excavated from soil cores taken 50–100 cm from the tree base in three directions, and adhering soil was gently removed. All organ samples (leaves, stems, and roots) from an individual tree were placed in separate, prelabeled kraft paper bags.

For enzyme activity assays, fresh subsamples of leaves, stems, and roots (approximately 0.5 g each) were immediately flash-frozen in liquid nitrogen in the field and subsequently stored at −80 °C until analysis.

For the analysis of stoichiometric traits and osmoregulatory substances, the remaining plant material was transported to the laboratory, oven-dried at 75 °C for 48 h to a constant weight, homogenized using a ball mill, and ground to a fine powder (passed through a 0.15 mm sieve).

### 4.4. Measurement of Plant Eco-Physiological Characteristics

Analyses were performed using the oven-dried, homogenized plant powder. All analyses were performed with three biological replicates and three technical replicates.

#### 4.4.1. Stoichiometric Traits

The plant organic carbon (POC) content was determined using the potassium dichromate oxidation method with external heating. Approximately 0.1 g of powder was digested with 5 mL of 0.8 mol/L K_2_Cr_2_O_7_ and 10 mL of concentrated H_2_SO_4_ at room temperature for 30 min. The excess dichromate was titrated with a standardized ferrous ammonium sulfate solution using phenanthroline as an indicator. The POC content was calculated from the difference in the titrant volume between the sample and a blank. The results are expressed on a dry weight basis (g/kg).

Plant total nitrogen (PN) was measured by the semimicro Kjeldahl method. Approximately 0.05 g of powder was digested with concentrated H_2_SO_4_ and H_2_O_2_. The digest was distilled, and the liberated ammonia was absorbed in boric acid and titrated with standard HCl. The PN concentration was calculated on the basis of acid consumption. The results are expressed on a dry weight basis (g/kg).

Plant total phosphorus (PP) was quantified colorimetrically [29]. An aliquot of the same digestate prepared for PN analysis was used. After neutralization, the phosphate was reacted with molybdenum–antimony–ascorbic acid to form a blue complex, the absorbance of which was measured at 880 nm using a UV–Vis spectrophotometer (UV-1900i; Shimadzu Corp., Kyoto, Japan). The PP concentration was determined using a KH_2_PO_4_ standard curve. The results are expressed on a dry weight basis (g/kg).

#### 4.4.2. Osmoregulatory Substances

The plant proline (Pro) content was assessed using the acid ninhydrin method [29]. Powder (0.3 g) was extracted with 5 mL of 3% (*w*/*v*) sulfosalicylic acid. A 2 mL aliquot of the extract was reacted with 2 mL each of glacial acetic acid and acid-ninhydrin reagent by heating at 100 °C for 30 min. The chromophore was extracted with toluene, and its absorbance was measured at 520 nm using a UV–Vis spectrophotometer (UV-1900i, Shimadzu Corp., Kyoto, Japan). The Pro concentration was determined using an L-proline standard curve and is expressed as µg per gram dry weight (µg/g).

The plant soluble protein (PSP) content was determined using the Coomassie Brilliant Blue G-250 binding method [29]. Proteins were extracted from 0.1 g of powder with 5 mL of cold 50 mM phosphate buffer (pH = 7.0). After centrifugation, a 0.1 mL aliquot of the supernatant was mixed with 5 mL of Coomassie Brilliant Blue reagent. The absorbance was measured at 595 nm after 2 min using a UV–Vis spectrophotometer (UV-1900i; Shimadzu Corp., Kyoto, Japan). The PSP concentration was determined using a bovine serum albumin (BSA) standard curve and is expressed as mg per gram dry weight (mg/g).

The plant soluble sugar (PSS) content was measured by the anthrone–sulfuric acid method [29]. Powder (0.1 g) was extracted twice with 5 mL of 80% (*v*/*v*) ethanol at 60 °C. An aliquot of the combined extract was reacted with anthrone reagent by heating at 100 °C for 10 min. Absorbance was measured at 620 nm using a UV–Vis spectrophotometer (UV-1900i; Shimadzu Corp., Kyoto, Japan). The PSS content was calculated using a glucose standard curve and is expressed as mg per gram dry weight (mg/g).

Plant starch (Sta) was analyzed by hydrolyzing the residue from the soluble sugar extraction [29]. The residue was boiled with water and 9.2 mol/L HClO_4_. The liberated glucose in the hydrolysate was quantified using the same anthrone method as that used for PSS, with the absorbance measured at 620 nm using a UV–Vis spectrophotometer (UV-1900i; Shimadzu Corp., Kyoto, Japan). The starch content was calculated as glucose equivalents multiplied by 0.9 and expressed as mg per gram dry weight (mg/g).

#### 4.4.3. Antioxidant Enzyme Activities

Assays were performed using fresh tissues. Approximately 0.5 g of fresh tissue (leaf, stem, or root) was homogenized in 4.0 mL of ice-cold 50 mM phosphate buffer (pH = 7.8) containing 1% (*w*/*v*) polyvinylpyrrolidone. The homogenate was centrifuged at 12,000× *g* for 20 min at 4 °C, and the resulting supernatant was used as the crude enzyme extract.

Superoxide dismutase (SOD) activity was determined by inhibiting the photoreduction of nitroblue tetrazolium (NBT) [29]. The 3 mL reaction mixture contained 50 mM phosphate buffer (pH = 7.8), 14.5 mM methionine, 30 µM EDTA-Na_2_, 60 µM riboflavin, and 2.25 mM NBT. The reaction was initiated by the addition of 30 µL of enzyme extract and illumination under 4000 lux light for 20 min. The absorbance was measured at 560 nm using a UV–Vis spectrophotometer (UV-1900i; Shimadzu Corp., Kyoto, Japan). One unit (U) of SOD activity was defined as the amount of enzyme required to inhibit 50% of NBT photoreduction. The activity was calculated as follows:

(1) $$\mathrm{SOD}\;\mathrm{activity}\;(U/g\;\mathrm{FW})\;=\left[\left(A_{0}\;-\;A_{S}\right)\right]/\left(0.5\;\times {\;A}_{0}\;\times \;W\;\times {\;V}_{S}\right)$$

where A_0_ and A_S_ are the absorbances of the control (without enzyme) and the sample, respectively; V_t_ is the total volume of the enzyme extract (mL); V_S_ is the volume of the enzyme extract used in the assay (mL); and W is the sample fresh weight (g).

Peroxidase (POD) activity was measured using guaiacol as a substrate [29]. The 3 mL reaction mixture contained 50 mM phosphate buffer (pH = 6.0), 20 mM guaiacol, and 40 mM H_2_O_2_. The increase in absorbance at 470 nm was recorded immediately after the addition of 30 µL of enzyme extract using a UV–Vis spectrophotometer (UV-1900i; Shimadzu Corp., Kyoto, Japan). Readings were taken every 10 s for 40 s. The change in absorbance per minute (ΔA470/min) was calculated from the linear portion of the curve. One unit (U) was defined as a change of 0.01 in absorbance per minute. The activity was calculated as follows:

(2) $$\mathrm{POD}\;\mathrm{activity}\;(U/g\;\mathrm{FW})\;=\;\left(∆A_{470}/\min\times \;V_{t}\right)/\left(W\;\times \;V_{S}\;\times \;0.01\right)$$

Catalase (CAT) activity was assayed by monitoring the decomposition of H_2_O_2_ at 240 nm [29]. The 3 mL reaction mixture contained 50 mM phosphate buffer (pH = 7.0) and 15 mM H_2_O_2_. The decrease in absorbance was recorded for 40 s after the addition of 100 µL of enzyme extract using a UV–Vis spectrophotometer (UV-1900i; Shimadzu Corp., Kyoto, Japan). The change in absorbance per minute (ΔA240/min) was calculated. One unit (U) was defined as a change of 0.01 in absorbance per minute. The activity was calculated as follows:

(3) $$\mathrm{CAT}\;\mathrm{activity}\;(U/g\;\mathrm{FW})\;=\;\left(∆A_{240}/\min\;\times {\;V}_{t}\right)/\left(W\;\times \;V_{S}\;\times \;0.01\right)$$

### 4.5. Soil Sample Collection, Processing, and Analysis

Soil sampling was conducted concurrently with plant collection. A composite soil sample per depth was created from five subsamples collected per plot using a five-point sampling method (four corners and the center). After surface litter was removed, soil cores were taken from three depths: 0–20 cm, 20–40 cm, and 40–60 cm. For soil water content determination, a separate undisturbed subsample from each depth was immediately sealed in a preweighed aluminum box. The remaining composite soil was air-dried, ground, and passed through a 1 mm sieve.

Soil pH and electrical conductivity (EC): Soil pH and EC were measured potentiometrically in a 1:2.5 (*w*/*v*) soil–water suspension for pH and a 1:5 (*w*/*v*) suspension for EC.

Soil water content (SWC): The SWC was determined gravimetrically by oven-drying the sealed aluminum boxes at 105 °C for 24 h to a constant weight.

Soil organic carbon (SOC): The SOC was analyzed using the potassium dichromate oxidation method (as described for POC).

Soil total nitrogen (SN) The SN was measured by the Kjeldahl method (as described for PN).

Soil total phosphorus (SP): The SP was quantified by the molybdenum–antimony colorimetric method after digestion with HClO_4_-H_2_SO_4_ (as described for PP).

### 4.6. Microenvironmental Conditions During Sampling Events

On each sampling day, microenvironmental variables were recorded at the center of each plot. During the period from 11:10 to 16:30 local time, measurements were taken simultaneously at 20 min intervals using the following instruments: light intensity (Illuminance, lux) was measured with a SHENDAWEI digital illuminance meter (Model SW-582, Manufacturer: Shenzhen Shendawei Technology Co., Ltd., Shenzhen, China); air temperature (AT, °C) and relative humidity (RH, %) were measured with a BIAOZHI digital thermohygrometer (Model GM1360A, Manufacturer: Shenzhen Biaozhi Instrument Co., Ltd., Shenzhen, China). For each variable, the average of all readings within this daily time window was calculated to represent the plot-specific microenvironmental condition for that specific sampling day. Solar radiation (SR, W/m^2^) was calculated from illuminance data using Equation (4). The vapor pressure deficit (VPD, kPa) was derived from the AT and RH using Equation (5):

(4) SR = 0.0104 × Lux

(5) $$\mathrm{VPD}=0.61078\;\times {\;e}^{\left(17.27\;\times \;\mathrm{AT}\right)/\left(\mathrm{AT}+237.3\right)}\;\times \;\left(1-\mathrm{RH}\right)$$

These measurements reflect the environmental conditions at the time of sampling and are not intended to represent seasonal or long-term climatic averages.

### 4.7. Data Analysis

The data were collated using Microsoft Excel 2013. Statistical analysis and graphing were performed using Origin 2018 and R (version 4.3.0). Descriptive statistics were used to calculate the means and standard deviations for all replicate groups. Prior to the parametric tests, all dataset groups were tested for normality using the Shapiro–Wilk test and for homogeneity of variance using Levene’s test. If the assumptions were violated, appropriate data transformations (e.g., log or square root) were applied. To examine the spatiotemporal differences, separate factorial ANOVAs were conducted. For the eco-physiological characteristics of *P. schrenkiana* (including stoichiometric traits, osmoregulatory substances, and antioxidant enzyme activities), three-way ANOVA was performed with the region, season, and plant organ as fixed factors. For soil physicochemical factors, three-way ANOVA was conducted with the region, season, and soil layer (depth) as fixed factors. For microenvironmental factors, two-way ANOVA (with region and season as factors) was employed. When ANOVA indicated significant effects (*p* < 0.05), post hoc multiple comparisons were conducted using Fisher’s LSD test. Associations among eco-physiological characteristics were determined by Pearson correlation analysis. To evaluate the integrated effects of environmental factors on the eco-physiological traits of *P. schrenkiana*, a partial least squares path model (PLS–PM) was constructed using the R package plspm (version 0.5.0). The model comprised seven latent variables: region (indicated by longitude and latitude), season (indicated by dummy variables for spring and summer), microenvironmental factors (indicated by relative humidity, air temperature, vapor pressure deficit, and solar radiation), soil factors (indicated by pH, soil organic carbon, soil nitrogen, and soil phosphorus), plant osmoregulatory substances (indicated by proline, soluble sugar, and starch contents), plant stoichiometric characteristics (indicated by organic carbon and phosphorus contents), and plant enzyme activity (indicated by superoxide dismutase and peroxidase activities). All latent variables were modeled as reflective constructs. The structural model specified that season and region directly influenced both microenvironmental factors and soil factors, with soil factors treated as endogenous variables. microenvironmental factors and soil factors in turn influenced the latent variables of the three plant traits, which were also intercorrelated. Path significance was assessed using bootstrap resampling with 500 iterations to generate bias-corrected 95% confidence intervals. Model fit was evaluated using the goodness-of-fit (GoF) index, with a value > 0.36 indicating acceptable global model fit. Path coefficients were considered statistically significant if their 95% confidence intervals did not cross zero. The significance level for all tests was set at α < 0.05.

## 5. Conclusions

This study elucidated the spatiotemporal dynamics and interrelationships of eco-physiological characteristics in *P. schrenkiana* across the Tianshan Mountains, revealing the physiological mechanisms underlying its environmental acclimation. The results demonstrated significant seasonal differences in eco-physiological characteristics (*p* < 0.05), with stoichiometric traits and osmolyte accumulation closely coupled to key antioxidant enzyme activities (*p* < 0.05). Under favorable spring conditions (i.e., optimal light and temperature), carbon allocation was prioritized to maximize photosynthesis to support rapid growth. During summer heat stress, resource partitioning shifted toward antioxidant defense and osmotic adjustment to mitigate reactive oxygen species damage. In preparation for winter freezing, autumn strategies emphasize nutrient reservoir formation through nitrogen resorption and carbon–nitrogen synergy. Soil factors were the primary positive regulators of enzymatic responses across seasons and elevations, whereas meteorological drivers exerted more complex, and often suppressive, influences. In conclusion, *P. schrenkiana* employs sophisticated spatiotemporal adaptation strategies by adjusting its eco-physiological characteristics in response to environmental changes mediated by soil and meteorological factors. Our findings elucidate the physiological mechanisms underlying the resilience of this key species in arid mountain ecosystems and provide a theoretical basis for its conservation under climate change conditions. Future research that integrates transcriptomic and molecular techniques could further reveal the genetic basis of these adaptive traits.

## Figures and Tables

**Table 1:** Geographical, topographic, and climatic traits of the three sampling stations.

Sampling Station and Abbreviation	Geographical Parameter			Topographic Parameters		
	Longitude (°E)	Latitude (°N)	Altitude (m)	Topographic Position	Aspect	Slope Gradient (°)
ETM	92°56′	43°32′	2052	Lower slope	North-facing	14
CTM	87°11′	43°29′	1990	Lower slope	North-facing	13
WTM	84°36′	44°7′	1992	Lower slope	North-facing	15

**Table 2:** Sample plant traits of the three sampling stations.

Sampling Station and Abbreviation	Mean Annual Meteorological Data			Plant Trait	
	Temperature (°C)	Precipitation (mm)	Relative Humidity (%)	DBH (cm)	H (m)
ETM	4.25	63	43.23	23.22 ± 2.60	8.08 ± 0.60
CTM	2.56	322	57.08	25.11 ± 0.21	9.40 ± 2.34
WTM	0.78	310	60.70	24.44 ± 2.41	9.86 ± 1.32

Climatic data were obtained from the NASA POWER database [27,28]. Values are mean ± SD of dominant trees in each plot. DBH: Diameter at Breast Height, H: tree height.

## Data Availability

The data presented in this study are available on request from the corresponding author due to restrictions on public data sharing. Requests for data access will be reviewed by the research team, and approved requests will be provided with the necessary datasets under a confidentiality agreement.

## Supplementary Materials

The following supporting information can be downloaded at https://www.mdpi.com/article/10.3390/plants15081199/s1. Table S1: Two-way analysis of variance evaluating seasonal and interregional effects on meteorological factors in *P. schrenkiana* forest ecosystems; Table S2: Seasonal microenvironmental conditions at the three sampling stations; Table S3: Results of the three-way analysis of variance (season × region × soil depth) for the soil factors of *P. schrenkiana*; Table S4: Three-way ANOVA of seasonal variations in stoichiometric characteristics of different organs of *P. schrenkiana* from various regions; Table S5: Results of three-way ANOVA on the effects of season and region on osmoregulatory substances in *P. schrenkiana* forest ecosystems; Table S6: Three-way ANOVA of seasonal variations in enzyme activity of different organs of *P. schrenkiana* from various regions; Table S7: Decomposition of effects showing direct, indirect, and total path coefficients in the partial least squares structural equation model.

## References

[1] Choat B., Brodribb T.J., Brodersen C.R., Duursma R.A., López R., Medlyn B.E. (2018). Triggers of tree mortality under drought. Nature.

[2] Pan Y., Birdsey R.A., Fang J., Houghton R., Kauppi P.E., Kurz W.A., Phillips O.L., Shvidenko A., Lewis S.L., Canadell J.G. (2011). A large and persistent carbon sink in the world’s forests. Science.

[3] Anderegg W.R.L., Klein T., Bartlett M., Sack L., Pellegrini A.F.A., Choat B., Jansen S. (2016). Meta-analysis reveals that hydraulic traits explain cross-species patterns of drought-induced tree mortality across the globe. Proc. Natl. Acad. Sci. USA.

[4] Song X., Li J., Zeng X. (2024). Parameterization of Tree and Shrub Stem Wood Density Adaptions to Multiple Climate and Soil Factor Gradients. Adv. Atmos. Sci..

[5] Liu Y., Nadezhdina N., Di N., Ma X., Liu J.Q., Zou S.Y., Xi B.Y., Clothier B. (2021). An undiscovered facet of hydraulic redistribution driven by evaporation—A study from a Populus tomentosa plantation. Plant Physiol..

[6] Dobbert S., Pape R., Löffler J. (2021). Contrasting growth response of evergreen and deciduousarctic-alpine shrub species to climate variability. Ecosphere.

[7] Buckley T.N. (2019). How do stomata respond to water status?. New Phytol..

[8] Wang C., Zhu K., Bai Y., Li C., Li M., Sun Y. (2024). Response of stomatal conductance to plant water stress in buffalograss seed production: Observation with UAV thermal infrared imagery. Agric. Water Manag..

[9] Jin Y., Ye Q., Liu X., Liu H., Gleason S.M., He P., Liang X., Wu G. (2024). Precipitation, solar radiation, and their interaction modify leafhydraulic efficiency–safety trade-off across angiosperms at theglobal scale. New Phytol..

[10] Peng F., Yuan M., Hou T., Zhou L., Zheng B., Wang Y. (2025). Identification and analysis of slow anion channel proteins (SLACs) gene families involved in drought stress in Orchidaceae. Ind. Crops Prod..

[11] Chen X., Xu W., Luo G., Lin Q., Xiao L. (2008). Soil properties at the tree limits of Picea schrenkiana forests in response to varying environmental conditions on the northern slope of Tianshan mountains. Acta Ecol. Sin..

[12] Tian S., Zhao S., Zheng X., Wang Y., Li Y. (2023). Water source of spruce (*Picea schrenkiana*) at different altitudes in the Tianshan Mountains during the growing season. Arid Zone Res..

[13] Zhao C., Bie Q., Peng H. (2010). Analysis of the niche space of Picea crassifolia on the northern slope of Qilian Mountains. Acta Geogr. Sin..

[14] Bao Q., Mu X., Tong C., Li C., Tao W., Zhao S., Liu Y., Wang W., Wei Y., Yu F. (2023). Sugar status in preexisting leaves determines systemic stomatal development within newly developing leaves. Proc. Natl. Acad. Sci. USA.

[15] Farrant J.M., Ruelland E. (2015). Plant signalling mechanisms in response to the environment. Environ. Exp. Bot..

[16] Poorter H., Niklas K.J., Reich P.B., Oleksyn J., Poot P., Mommer L. (2012). Biomass allocation to leaves, stems and roots: Meta-analyses of interspecific variation and environmental control. New Phytol..

[17] Suzuki N., Koussevitzky S., Mittler R., Miller G. (2012). ROS and redox signalling in the response of plants to abiotic stress. Plant Cell Environ..

[18] Zandalinas S.I., Mittler R., Balfagón D., Arbona V., Gómez-Cadenas A. (2018). Plant adaptations to the combination of drought and high temperatures. Physiol. Plant..

[19] Kökdener M., Yurtgan M.S. (2022). The Effect of Soil Type and Moisture Level on the Development of Lucilia sericata (Diptera: Calliphoridae). J. Med. Entomol..

[20] Hou X., Dan Y., Qi N., Zhang M., Li C., Li Y., Yao Y., Liao W. (2024). Nitric Oxide Promotes Adventitious Rooting in Cucumber Under Drought Stress Through Regulating Ascorbate Glutathione Cycle and Protein S-Nitrosylation. J. Plant Growth Regul..

[21] Emna G., Khaled S., Moez J., Yassine H., Rim N.O., Yordan M., Salwa H.J., Mohamed E.A., Souhir A., Oumaima C. (2021). Physiological responses and expression of sugar associated genes in faba bean (*Vicia faba* L.) exposed to osmotic stress. Physiol. Mol. Biol. Plants.

[22] Liu J., Lyu M., Xu X., Liu C., Qin H., Tian G., Zhu Z., Ge S., Jiang Y. (2022). Exogenous sucrose promotes the growth of apple rootstocks under high nitrate supply by modulating carbon and nitrogen metabolism. Plant Physiol. Biochem..

[23] Zhang K., Jiao L., Xue R., Zhang P., Yuan X., Wang X., Li Q., Guo Z., Qing Y., Zhang L. (2025). Elevation pattern of resource allocation in Picea crassifolia Kom. and its coupling mechanism with soil factors in Helan Mountains, China. AoB Plants.

[24] Liu J., Guo X., Wang F., Liu J., Zhang F. (2023). Seasonal patterns in the leaf C:N:P stoichiometry of four conifers on the northeastern Tibetan Plateau. Glob. Ecol. Conserv..

[25] Feng Z., Zheng F., Wu S., Li R., Li Y., Zhong J., Zhao H. (2021). Functional Characterization of a Cucumber (*Cucumis sativus* L.) Vacuolar Invertase, CsVI1, Involved in Hexose Accumulation and Response to Low Temperature Stress. Int. J. Mol. Sci..

[26] Peng Z., Zhang Y., Zhu L., Guo M., Lu Q., Xu K., Shao H., Mo Q., Liu S. (2023). Spatial and temporal patterns of the sensitivity of radial growth response by Picea schrenkiana to regional climate change in the Tianshan Mountains. J. For. Res..

[27] NASA Langley Research Center (2024). NASA Prediction of Worldwide Energy Resources (POWER) Project. https://power.larc.nasa.gov/.

[28] Stackhouse P.W., Westberg D., Hoell J.M., Chandler W.S., Zhang T. (2018). NASA Prediction of Worldwide Energy Resources (POWER) Project Data Methodology and Versions.

[29] Li H.S., Li H.S. (2000). Determination of proline content by acidic ninhydrin method. Principles and Techniques of Plant Physiological and Biochemical Experiments.

