# Supplementary material for: Spatiotemporal Dynamics of the Eco-Physiological Characteristics of Picea schrenkiana in the Tianshan Mountains and Its Adaptive Mechanisms
Source: Plants (Basel). 2026 Apr 14;15(8):1199. doi: 10.3390/plants15081199 (PMC13119731; doi:10.3390/plants15081199)
Supplement: Supplementary file 1 [file plants-15-01199-s001.zip › plants-4227632-supplementary.pdf]

Supplementary Material

Spatiotemporal Dynamics of Eco-physiological Characteristics in *Picea schrenkiana* on the Tianshan Mountains and Its Adaptive Mechanisms

Ruixi Li [a, b, c]; Lu Gong [a, b, c, *]; Xue Wu [a, b, c]; Kejie Yin [a, b, c]; Yihu Niu [a, b, c]; Xiaonan Sun [a, b, c]; Peryzat Abay [a, b, c]; Fan Tian [a, b, c]

a College of Ecology and Entironment ,Xinjiang University, Urumqi, China

b Key Laboratory of Oasis Ecology of Education Ministry, Urumqi, China

c Xinjiang Jinghe Observation and Research Station of Tenperate Desert Ecosystem, Ministry of Education, Jinghe, China

Table S1 Two-way analysis of variance evaluating seasonal and interregional effects on microenvironmental factors in *P. schrenkiana* forest ecosystems

Tested effects	SR		RH		AT		VPD	
	F	*P*	*F*	*P*	*F*	*P*	*F*	*P*
Seasons	2.414	0.097	44.686	< 0.001 **	336.858	< 0.001 **	127.079	< 0.001 **
Regions	59.413	< 0.001 **	49.338	< 0.001 **	47.763	< 0.001 **	9.538	< 0.001 **
Season*regions	17.703	< 0.001 **	2.880	0.029 *	7.753	< 0.001 **	2.576	0.045 *

solar radiation (SR), relative humidity (RH), air temperature (AT), and vapor pressure deficit (VPD). **: *P* < 0.001; *: *P* < 0.05.

Table S2. Seasonal microenvironmental conditions at the three sampling stations.

Seasons	Regions	SR (W/m^2)	RH (%)	AT (°C)	VPD (kPa)
spring	ETM	948.56 ± 127.62	35.78 ± 0.92	12.06 ± 0.42	-48.94 ± 1.40
	CTM	196.85 ± 23.55	52.44 ± 2.64	7.48 ± 1.18	-54.58 ± 5.47
	WTM	185.38 ± 38.67	54.27 ± 3.44	5.07 ± 0.29	-46.93 ± 3.41
summer	ETM	680.30 ± 143.05	21.49 ± 0.74	31.11 ± 1.05	-93.22 ± 5.92
	CTM	996.19 ± 162.66	37.38 ± 0.79	23.87 ± 0.24	-107.53 ± 1.26
	WTM	103.21 ± 33.49	53.77 ± 6.04	19.26 ± 1.37	-114.69 ± 8.58
autumn	ETM	1172.16 ± 64.85	45.33 ± 5.05	10.11 ± 1.27	-52.78 ± 3.71
	CTM	89.27 ± 46.78	72.51 ± 3.15	7.18 ± 0.87	-71.87 ± 1.22
	WTM	133.47 ± 22.20	70.04 ± 1.93	8.16 ± 0.35	-74.64 ± 0.73

solar radiation (SR), relative humidity (RH), air temperature (AT), and vapor pressure deficit (VPD).

[*] Corresponding author at: College of Ecology and Environment, Xinjiang University, Urumqi, China.
E-mail address: gonglu721@163.com (L. Gong).

Table S3. Results of the three-way analysis of variance (season × region × soil depth) for the soil factors of *P. schrenkiana.*

Tested effects	SWC		EC		pH		SOC		SN		SP	
	F	*P*	*F*	*P*	*F*	*P*	*F*	*P*	*F*	*P*	*F*	*P*
Seasons	0.843	0.436	6.996	0.002*	14.057	< 0.001**	5.505	0.007*	37.312	< 0.001**	148.384	< 0.001**
Regions	3.729	0.030*	1.540	0.224	30.391	< 0.001**	4.586	0.014*	27.919	< 0.001**	1.245	0.296
Depth	15.592	< 0.001**	0.536	0.588	5.354	0.008*	12.441	< 0.001**	38.010	< 0.001**	2.450	0.096
Season*regions	0.971	0.431	1.046	0.392	3.234	0.019*	0.744	0.566	8.750	< 0.001**	4.031	0.006*
Season*depth	0.950	0.442	0.888	0.477	2.845	0.033*	0.114	0.977	5.571	< 0.001**	8.730	< 0.001**
Regions*depth	1.867	0.130	0.869	0.489	0.779	0.544	1.272	0.292	8.564	< 0.001**	2.753	0.037*
Season*regions*depth	0.208	0.988	0.438	0.893	2.109	0.051	0.280	0.970	2.968	0.008*	2.969	0.008*

SWC: soil water content; EC: soil conductivity ; SOC: soil organic carbon; SN: soil total nitrogen; SP: soil total phosphorus. **: $P < 0.001$; *: $P < 0.05$.

Table S4. Three-way ANOVA of seasonal variations in stoichiometric characteristics of different organs of *P. schrenkiana* from various regions.

Tested effects	POC		PN		PP	
	F	*P*	*F*	*P*	*F*	*P*
Seasons	36.699	< 0.001**	42.965	< 0.001**	4.648	0.014*
Regions	0.172	0.843	10.586	< 0.001**	4.542	0.015*
Organs	5.159	0.009*	72.205	< 0.001**	1.630	0.205
Season*regions	2.162	0.086	1.602	0.187	1.191	0.325
Season*organs	0.925	0.456	2.784	0.04*	3.241	0.019*
Regions*organs	4.777	0.002*	6.873	< 0.001**	0.314	0.868
Season*regions*organs	1.308	0.260	3.251	0.004*	1.689	0.122

**: $P < 0.001$; *: $P < 0.05$. POC: plant organic carbon; PN: plant total nitrogen; PP: plant total phosphorus. Sampling rounds correspond to spring, summer, and autumn sampling dates, respectively.

Table S5. Results of three-way ANOVA on the effects of season and region on osmoregulatory substances in *P. schrenkiana* forest ecosystems.

Tested effects	Pro	PSP	PSS	Sta

	F	*P*	*F*	*P*	*F*	*P*	*F*	*P*
Seasons	6.089	0.004*	7.458	0.001*	159.432	< 0.001**	42.894	< 0.001**
Regions	4.306	0.019*	0.655	0.523	49.696	< 0.001**	0.440	0.646
Organs	10.871	< 0.001**	28.763	< 0.001**	476.723	< 0.001**	50.877	< 0.001**
Season*regions	8.141	< 0.001**	4.124	0.005*	2.660	0.043*	0.705	0.592
Season*organs	5.747	< 0.001**	1.726	0.158	17.590	< 0.001**	6.589	< 0.001**
Regions*organs	15.518	< 0.001**	3.423	0.014*	7.003	< 0.001**	2.297	0.071
Season*regions*organs	9.107	< 0.001**	1.217	0.307	7.067	< 0.001**	3.881	0.001*

**: *P* < 0.001; *: *P* < 0.05. Pro: proline; PSP: soluble protein; PSS: soluble sugar; and Sta: starch.

Table S6. Three-way ANOVA of seasonal variations in enzyme activity of different organs of *P. schrenkiana* from various regions.

Tested effects	SOD		POD		CAT	
	F	*P*	*F*	*P*	*F*	*P*
Seasons	226.050	< 0.001**	1050.222	< 0.001**	5.183	0.009*
Regions	46.496	< 0.001**	13.986	< 0.001**	0.027	0.973
Organs	1418.455	< 0.001**	1189.058	< 0.001**	64.655	< 0.001**
Season*regions	3.946	0.007*	13.559	< 0.001**	2.743	0.038*
Season*organs	48.623	< 0.001**	271.426	< 0.001**	57.518	< 0.001**
Regions*organs	31.433	< 0.001**	14.479	< 0.001**	3.955	0.007*
Season*regions*organs	1.951	0.071	16.302	< 0.001**	1.392	0.221

**: *P* < 0.001; *: *P* < 0.05. SOD: superoxide dismutase; POD: peroxidase; CAT: catalase.

Table S7. Decomposition of effects showing direct, indirect, and total path coefficients in the partial least squares structural equation model.

Relationships	Direct Effect	Indirect Effect	Total Effect	Significance
Season -> Meteorological factors	0.764	0	0.764	
Region -> Meteorological factors	-0.429	0	-0.429	
Season -> Soil factors	0.629	-0.009	0.62	

Region -> Soil factors	-0.365	0.005	-0.359	***
Meteorological factors -> Soil factors	-0.012	0	-0.012	
Meteorological factors -> Plant osmoregulatory substances	0.079	-0.003	0.076	
Soil factors -> Plant osmoregulatory substances	0.218	0	0.218	
Meteorological factors -> Plant stoichiometric characteristics	-0.17	0.053	-0.117	
Soil factors -> Plant stoichiometric characteristics	-0.369	0.139	-0.23	***
Plant osmoregulatory substances -> Plant stoichiometric characteristics	0.637	0	0.637	***
Meteorological factors -> Plant enzyme activity	-0.038	0.04	0.002	
Soil factors -> Plant enzyme activity	0.517	0.123	0.64	***
Plant osmoregulatory substances -> Plant enzyme activity	0.49	-0.046	0.444	***
Plant stoichiometric characteristics -> Plant enzyme activity	-0.072	0	-0.072	

* $p < 0.05$, ** $p < 0.01$, *** p < 0.001
